# “The Disease Awareness Innovation Network” for chronic kidney disease identification in general practice

**DOI:** 10.1007/s40620-022-01353-6

**Published:** 2022-06-14

**Authors:** Francesco Pesce, Domenico Pasculli, Giuseppe Pasculli, Luca De Nicola, Mario Cozzolino, Antonio Granata, Loreto Gesualdo

**Affiliations:** 1 Nephrology, Dialysis, and Transplantation Unit, Department of Emergency and Organ Transplantation, University of Bari Aldo Moro, Azienda Ospedaliero Universitaria Consorziale Policlinico di Bari, Piazza Giulio Cesare, 11, 70124 Bari, Italy; 2 ASL BA-SIMG Societa’ Italiana Medici di Medicina Generale e delle Cure Primarie, Florence, Italy; 3 Department of Computer, Control and Management Engineering Antonio Ruberti (DIAG), La Sapienza University, Rome, Italy; 4 Division of Nephrology, University of Campania “Luigi Vanvitelli”, Naples, Italy; 5 Renal Division and Laboratory of Experimental Nephrology, Department of Health Sciences, University of Milan, Milan, Italy; 6 Nephrology and Dialysis Unit, “Cannizzaro” Hospital, 95126 Catania, Italy

**Keywords:** Chronic kidney disease, Awareness, Primary care, Early diagnosis

## Abstract

**Background:**

The “awareness gap” and the under-recognition of chronic kidney disease (CKD) by general practitioners (GPs) is well documented. We set a framework to evaluate the impact in primary care of targeted training and networking with nephrologists with regard to CKD awareness in terms of potential increase of the proportion of patients classified according to KDIGO in the general population and in patients with diabetes, hypertension and heart failure.

**Methods:**

Data were extracted from the Millewin Digital Platform in use by the GPs (*N* = 17) at baseline (T0, *N* = 17,854) and after 6 months (T6, *N* = 18,662) of networking (education, instant messaging and selected joint visits) with nephrologists (*N* = 2). The following variables were extracted: age, sex, eGFR (estimated glomerular filtration rate), ACR (urinary albumin-to-creatinine ratio), presence of type 2 diabetes, hypertension and heart failure. The proportion of patients detected having an eGFR below 60 mL/min/1.73m^2^ was also reported as deemed clinically relevant.

**Results:**

We observed an increase in the use of ACR and eGFR tests in the entire cohort (+ 121% and + 73%, respectively) and in patients with comorbidities. The proportion of patients with eGFR < 60 mL/min/1.73m^2^ significantly increased from 2.2% to 3.8% in the entire cohort,  from 6.3% to 12.7% in patients with diabetes, and from 5.6% to 9.9% in those with hypertension and finally from 10.8% to 23.7% in patients with heart failure.

**Conclusions:**

Training and network support to GPs by nephrologists can improve CKD awareness and increase its identification in the general population and, even more, in categories at risk.

**Graphical abstract:**

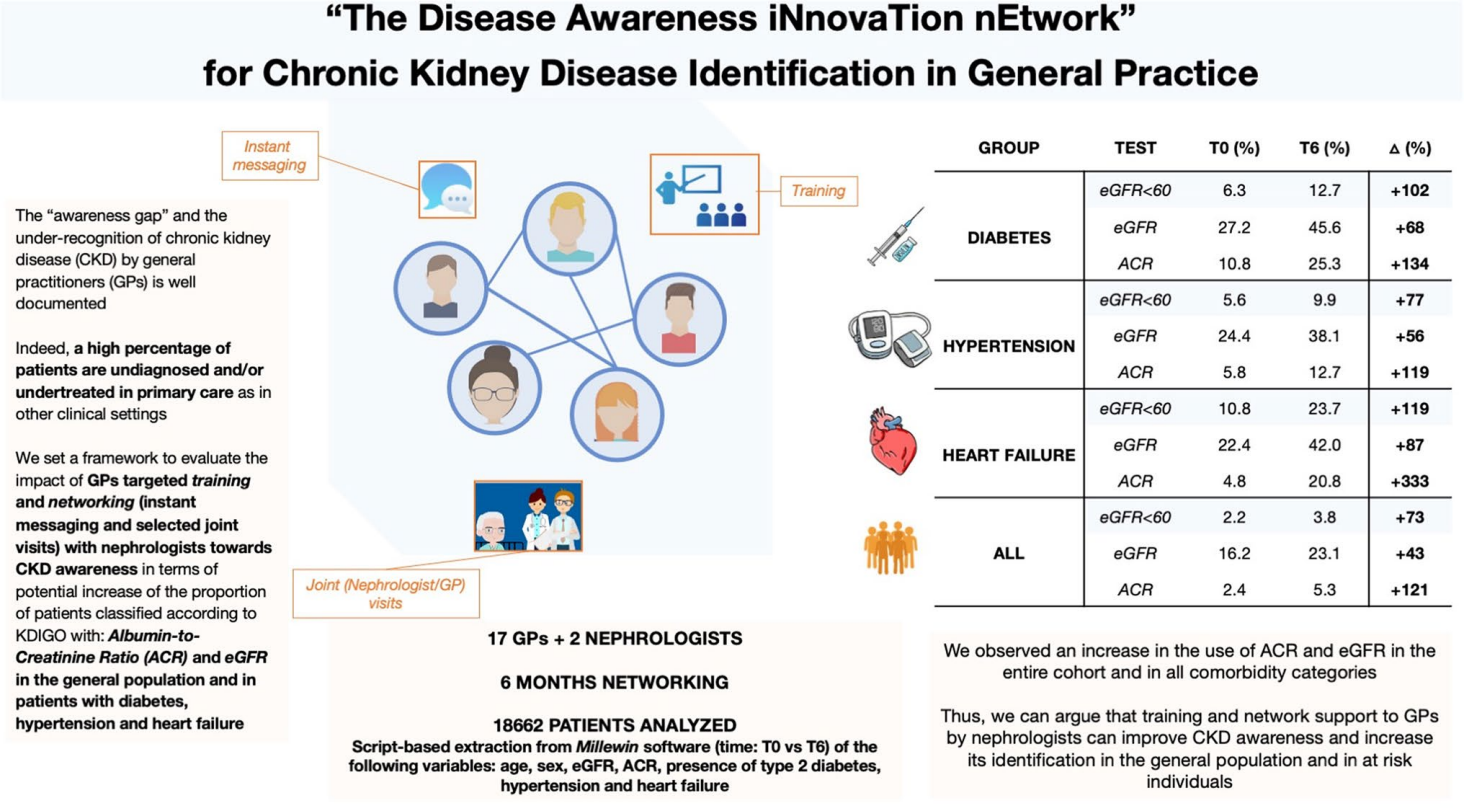

**Supplementary Information:**

The online version contains supplementary material available at 10.1007/s40620-022-01353-6.

## Introduction

The global burden of chronic kidney disease (CKD) is growing, impacting between 3 and 18% of the population worldwide [1]. CKD occurs when structural or functional kidney damage persists for longer than three months and it is characterised by a progressive reduction in the glomerular filtration rate (GFR) along with alterations of other biomarkers, such as the Albumin-to-creatinine ratio (ACR). The Kidney Disease Improving Global Outcomes (KDIGO) initiatives provided a risk classification for general outcomes based on the estimated glomerular filtration rate (eGFR) and ACR and specific recommendations on early identification and treatment of CKD [2].

As for other non-communicable and infectious diseases, early intervention is critical for lowering CKD-related morbidity and death [3]. The fact that the early stages of CKD are generally asymptomatic, however, challenges clinical guideline recommendations for early detection of CKD. Indeed, a high percentage of patients are undiagnosed and/or undertreated in primary care as in other clinical settings [4–6]. As a result, high-risk individuals, such as those with hypertension (HT), diabetes (T2DM, obesity, cardiovascular disease and a family history of renal disease, require targeted and accessible screening.

More than 850 million people worldwide suffer from CKD and, in 2030, CKD is expected to become the fifth leading cause of death in the world [7]. CKD has a prevalence of about 7% in Italy, with 8.1% in male and 7.8% women, and differences amongst northern-cental-southern Italian macro areas [8–10].

In a study promoted by the Italian Society of Nephrology (SIN) and the Italian Society of General Practitioners (SIMG) [11], 300 general practitioners (GPs) recruited a cohort of about 500,000 patients in the general population, showing that creatinine dosage had been requested for only 17% of patients. Of this subgroup, 16% of individuals were affected by CKD (eGFR < 60 mL/min/1.73m^2^), but among them, only 1 out of 8 patients had actually been identified by the GP as nephropathic, stressing the potential harm to the patients due to a possible missed diagnosis. Furthermore, such study highlighted that in Italy a nephrological consultation is usually requested in only 5% of patients with overt nephropathy in the conservative phase (eGFR 30–60 mL/min/1.73m^2^), whereas nephrological referral did not exceed 50% in cases of pre-dialysis disease (eGFR 30–15 mL/min/1.73m^2^). Similarly, low awareness of CKD has been reported for hypertensive patients attending Italian primary care offices; in a representative sample of adult hypertensive patients regularly followed up by a GP (*n* = 39,525), creatinine testing was reported for 59% of the patients, and a diagnosis of CKD was correctly reported in only 14% of them [12].

Furthermore, in 2018, the IRIDE (Italian obseRvatIonal study on management of CKD patiEnts and related costs) described the first Italian prospective, multicenter, observational study on the course of kidney disease and the clinical management of subjects over a 3-year period [13]. The management of CKD in clinical practice is essential for reducing disease progression and for providing improved control of secondary diseases. Findings from the IRIDE study indicate that greater attention is required for the control of renal function and proteinuria.

CKD in Italy is characterised by a lower prevalence compared to other western countries, but by a higher cardiovascular risk, attributable at least in part to the more advanced age, compared to subjects without kidney disease. A very interesting datum also concerns the greater prevalence of the earlier stages of CKD (1 and 2) [14]. Hence, there is high need for an earlier diagnosis in this subgroup of patients towards a more precise diagnosis of kidney disease and timely treatment. Renal replacement treatment for end stage renal disease (ESRD), which represents the natural evolution of CKD if left untreated, currently represents a substantial burden for the health care system [15]. From the health care system perspective, progression from CKD stages 1–2 to CKD stages 3a–3b was associated with a 1.1- to 1.7-fold increase in per patient mean annual health care cost [16].

Therefore, despite the relatively low prevalence, 1.8% of the total budget for health care is spent for ESRD patients, representing only 0.083% of the general population and amounting to a total of 2.5 billion euro [17]. Szczech A et al. [18] conducted a multicentre observational study assessing CKD prevalence in the adult population. They enrolled 9,339 patients through 466 investigator sites. The authors found that recommended urine CKD testing is underused in primary care, and that CKD is significantly underdiagnosed. CKD can be identified using two easy and inexpensive tests (eGFR from blood and ACR from urine), and patients at risk should be enrolled in cost-effective CKD early detection programs. People with obesity, diabetes, hypertension, heart disease, and a family history of CKD are at elevated risk of developing the condition, and depending on the region, comorbidities, environmental exposure, and genetic risk factors may also be relevant to consider [19]. Despite this, only 3% of health expenditure is invested in prevention, of which only 20% for early diagnosis.

Given the global shortage of CKD specialists, educating GPs and building successful multidisciplinary teams to play a larger role in early diagnosis and management would help to alleviate the CKD burden in hospitals and health care systems. Thus, the primary aim of this project was to evaluate the impact of targeted training for GPs by specialised nephrologists with regard to CKD awareness in terms of potentially increasing the proportion of patients tested for specific CKD biomarkers: ACR and eGFR. A single-arm, non randomized, paired proof of concept (POC) study was undertaken between 01/05/2021 and 31/10/2021 in Apulia region, Italy, within a framework called “The Disease Awareness Innovation Network (DANTE)”.

## Methods

### Study design

The primary endpoint of this single-arm, non randomized, paired proof of concept study was to evaluate the impact in primary care of targeted training and networking with nephrologists with regard to CKD awareness in terms of potential increase in the proportion of patients classified according to KDIGO in the general population and after stratifying patients by the following comorbidities: diabetes, hypertension and heart failure.

**Fig. 1:**
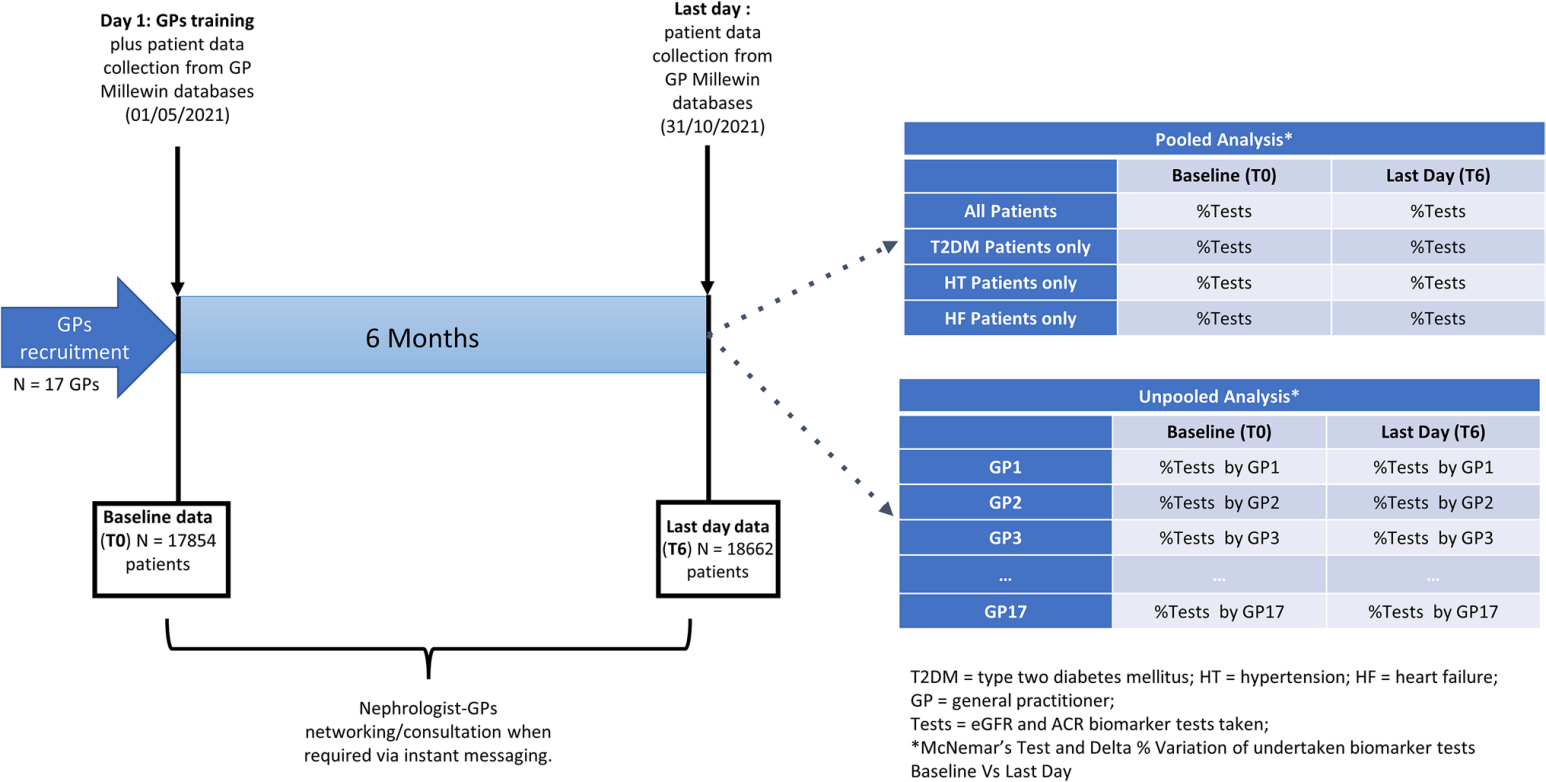
Study design and Statistical analyses scheme

A schematic representation of the study design along the statistical analyses undertaken is provided in Fig. 1.

### General practitioner training and networking

A first meeting was set up with GPs in which two trained nephrologists illustrated the KDIGO CKD guidelines to the GPs. In order to facilitate the exchange of information between the specialists and the GPs, an instant messaging chat was created after the beginning of the study allowing for mobile communication and discussion of specific cases. Joint visits (nephrologist and GP) were planned during the 6-month networking period. A final meeting was also planned in order to evaluate the effect of CKD awareness/specialist training after 6 months (T6) in terms of proportion of new CKD diagnostic tests (ACR, eGFR) undertaken, hence providing the GPs with a preliminary result of the impact of CKD awareness on their daily practice/patients.

### Study data

Data considered for the study were collected from the whole cohort of patients stored within the Millewin Digital Platform in use by the GPs included in the study (*n* = 17**,** all located in Apulia region, Italy). Data were analysed for the presence of specific comorbidities (type 2 diabetes mellitus, hypertension and heart failure) and for the proportion of ACR and eGFR (CKD-EPI formula) tests undertaken at baseline (T0) and after 6 months (T6).

In particular, we extracted the following variables: age, sex, eGFR, ACR, comorbidities. The proportion of patients whose eGFR was below 60 mL/min/1.73m^2^ was also reported as this information was deemed clinically relevant for the study.

### Millewin computerized medical record

The data considered for the study were collected by extraction from the Millewin computerized medical record: a computerized and problem-oriented medical record (CMOP), equipped with a Clinical Decision Support System (DSS), and a software (Milleutilità) based on the Postgres-SQL language for the extraction of recorded data, used by the general practitioners included in the study (*n* = 17, residing in Apulia region, Italy) in their usual clinical practice. Millewin is a Class 1 (as per EU 2017/745 regulation^1^) Medical Device (Medical devices are all instruments, appliances, equipment, software, implants, reagents, materials or other items that are used alone or in combination to intervene on a subject, i.e. human being, and called a patient for diagnosis, prevention, monitoring, prediction disease, prognosis, treatment) registered with the Italian Ministry of Health with ID 1847935. In order to guarantee homogeneity of data collection/management and results, only Apulian GPs with Millewin digital platform installed were selected for this study. Participating GPs were provided with a script SQL (Supplementary Material S1) to standardise the extraction of relevant data from the medical record using the software Milleutilità. Patients’ data were anonymized. All entries were used for the statistical analyses.

### Statistical analyses

A significance level of *α* = 5% was specified before data analysis. All patients collected by the 17 participating GPs were pooled into two main groups (baseline time [T0] vs time at six months [T6]) for the purpose of the main analyses.

For the descriptive analysis part: the difference in the numerical continuous covariate (age) between the two pooled patient groups (T0 and T6) was evaluated using a Wilcoxon two-sided test after a Shapiro–Wilk Test, plus quantile–quantile plots were conducted for normality evaluation of such covariate. For categorical variables (i.e. sex plus our collected diseases: HF, HT and T2DM), a McNemar’s Chi Squared test was used to test whether there was a difference in proportions between the two groups i.e., T0 vs T6.

For the primary endpoint analysis part: The Delta (variation in terms of % of diagnostic tests undertaken by the 17 GPs participating in the study between T0 and T6 pooled patients) was calculated as $\frac{{\text{\%}}_{T6}-{\text{\%}}_{T0}}{{\text{\%}}_{T0}}\times 100.$ The same Delta evaluation was also conducted after stratifying patients by their diseases (HF, HT and T2DM). The comparison was made using a two-sided McNemar’s test, with a Type I error rate (α) of 0.05. To detect a McNemar with an effect size (odds ratio) of 2.5 with a sample size of 200 observations, the power was estimated to be 0.92. The details of the previous steps can be found in Supplementary Materials 2 and 3.

As further validation, the latter analysis was also conducted by single GPs as statistical units instead of pooling patients collected by all 17 GPs by the two observation times (T0 and T6). The latter analysis is detailed in Supplementary Material 4**.** All analyses were conducted in R version 4.0.5 (2021-03-31) running under Windows 11 × 64 and schematically represented in Fig. 1 in the context of the study design.

## Results

A total of 17,854 patients, whose characteristics are shown in Table 1, were analysed at T0 (baseline) by retrieving data from the databases of 17 Apulian GPs who fulfilled the entry criterion (having Millewin software installed on their patient management systems). Mean age was 52.68 (20.5) years with a proportion of 50.6% of females.

**Table 1:** differences between T0 and T6 (pooled patients) in terms of collected covariates

Covariate	T0 (*N* = 17,854)	T6 (*N* = 18,662)	*p* value
Age			0.467^a^
Count	17,854	18,662	
Mean (SD)	52.66 (20.55)	52.80 (20.54)	
Sex			0.747^b^
M	8826 (49.4%)	9257 (49.6%)	
T2DM			0.316^b^
Cases	1932 (10.8%)	1959 (10.5%)	
HT			0.159.^2^
Cases	5878 (32.9%)	6015 (32.2%)	
HF			0.470^b^
Cases	250 (1.4%)	245 (1.3%)	

*T2DM* type 2 diabetes mellitus, *HT* hypertension, *HF* heart failure, *SD* standard deviation

^a^Wilcoxon two-sided test

^b^McNemar's Chi-squared test

Diabetics, hypertensives and patients with a diagnosis of heart failure account for 10.8%, 32.9% and 1.4%, respectively, of the entire cohort. Although the number of individuals at the end of the 6-month observation period (T6) was higher (*n* = 18,862), likely due to the fact that these GPs received additional patients from retired colleagues, we did not find any statistical difference in the prevalence of these categories of disease. This allowed us to properly measure the efficacy of the intervention, to use eGFR and ACR as basic diagnostic tools of CKD 6 months after training the GPs.

As for the pooled analysis results in Table 2, at T6 (after training the GPs) we observed a percentile increase of 43% (from 16.2 to 23.1%) in the use of eGFR and of 121% (from 2.4 to 5.3%) for ACR in the overall cohort. The same positive trend was observed after stratifying the pooled cohort for all the disease categories taken into account. In particular, for diabetic patients, the use of eGFR increased from 27.2 to 45.6% (+ 68%) and ACR went from 10.8 to 25.3% (+ 134%). The percentage of hypertensive patients screened for eGFR and ACR increased from 24.4 to 38.1% (+ 77%) and from 5.8 to 12.7% (+ 119%), respectively. In patients with heart failure, the use of eGFR showed an increase of 87% (from 22.4 to 42%) and for ACR of 333% (from 4.8 to 20.8%).

**Table 2:** Variations in diagnostic tests prescribed between T0 (Baseline) and T6 (6 months after GP training)

Group	Variable	T0 (%)	T6 (%)	Delta (%) ^a,b^
T2DM	eGFR < 60 mL/min/1.73m^2^	6.3	12.7	+ 102
	eGFR	27.2	45.6	+ 68
	ACR	10.8	25.3	+ 134
HT	eGFR < 60 mL/min/1.73m^2^	5.6	9.9	+ 77
	eGFR	24.4	38.1	+ 56
	ACR	5.8	12.7	+ 119
HF	eGFR < 60 mL/min/1.73m^2^	10.8	23.7	+ 119
	eGFR	22.4	42.0	+ 87
	ACR	4.8	20.8	+ 333
Overall^c^	eGFR < 60 mL/min/1.73m^2^	2.2	3.8	+ 73
	eGFR	16.2	23.1	+ 43
	ACR	2.4	5.3	+ 121

*T2DM* type 2 diabetes mellitus, *HT* hypertension, *HF* heart failure

^a^Calculated as $$\frac{{\text{\%}}_{T6}-{\text{\%}}_{T0}}{{\text{\%}}_{T0}}\times 100$$

^b^McNemar’s *χ*^2^ test for proportions, *p* < 0.0001

^c^Pooled patients (not stratified by disease group)

These results also allowed to increase the prevalence of individuals correctly diagnosed with CKD stages 3 to 5 (eGFR < 60 mL/min/1.73m2), which in the overall cohort reached 3.8% at T6 compared to the initial 2.2% at T0, with a detection percentage increase of 73% after the intervention (training) on the awareness of GPs for CKD. Likewise, in the subset of diabetic patients we observed a 101% increase in CKD stages 3 to 5 diagnoses (from 6.3 to 12.7%). For hypertensive patients and those with heart failure this prevalence increased from 5.6 to 9.9% (+ 77%) and 10.8 to 23.7% (119%), respectively. All of these comparisons, in terms of delta of prevalence of such tests between T0 and T6 (pooled analysis), yielded a statistically significant *p* value (< 0.0001).

Finally, in Table 3 we show patients classified according to the KDIGO, hence using both eGFR and ACR as available at the end of the observation period in the entire cohort. This table highlights that most individuals followed up by GPs are classified as A1 (89.5%) and 9.1% are classified as A2, whereas, in terms of eGFR, the large majority are G1-G3a (90.9%).

**Table 3:** Prognosis of CKD by GFR and albuminuria categories (KDIGO 2012) classification into subgroups at T6 (*n* = 849 patients after stratification by T6, eGFR and ACR tests taken)

	Persistent albuminuria categories, description and range							
	A1		A2		A3		Total (%)	
	Normal to mildly increased		Moderately increased		Severely increased			
	< 30 mg/g < 3 mg/mmol		30–300 mg/g 3–30 mg/mmol		> 300 mg/g > 30 mg/mmol			
GFR categories (ml/min/1.73 m^2^) description and range								
G1								
Normal or high								
≥ 90	179		15		3		197	23.2%
G2								
Mildly decreased								
60–89	409		32		2		443	52.2%
G3a								
Mildly to moderately decreased								
45–59	119		13		0		132	15.5%
G3b								
Moderately to severely decreased								
30–44	34		14		4		52	6.1%
G4								
Severely decreased								
15–29	16		1		3		20	2.4%
G5								
Kidney failure								
< 15	3		2		0		5	0.6%
Total (%)	760	89.5%	77	9.1%	12	1.4%	849	100.00%

## Discussion

The “awareness gap” and the under-recognition of CKD by GPs is well documented [20], and the need to demonstrate that the identification and treatment of early CKD truly impacts care is more urgent now than ever.

However, according to van Dipten C et al. [21] GPs might have differing opinions on how to define CKD and interpret eGFR. Despite the essential role of the quantification of urinary albumin and protein in the assessment of CKD, the low implementation of this test by GPs is a common problem globally [20, 22, 23], suggesting that a formal strategy for knowledge translation should be developed and that it should be based on educational tools, measurement of the effectiveness of such tools, and clear and consistent messaging.

The DANTE project takes these steps exactly, and in particular frames the CKD interventions specifically, within the context of cardiovascular health and diabetes, as suggested in order to effectively improve the management of CKD in primary care [24].

Of note, the use of iconic cases for discussion during the planned meetings with the GPs at T0 as well as at T6 was particularly appreciated. Even more efficient was the setup of a shared group (all GPs and the trained nephrologists) on an instant messaging platform. In fact, this allowed the management of specific real-life cases suggested by the GPs that were particularly challenging in terms of multidisciplinarity. Moreover, thanks to this close interaction, we identified patients who then underwent a renal biopsy that revealed glomerulonephritis and were treated timely.

In the DANTE pilot study, the first of its kind ever planned in Italy, we quantitatively evaluated the impact of such specialized training with regard to the GPs’ CKD awareness in terms of potential variation (% Delta) of diagnostic tests (eGFR and ACR) prescribed by GPs to their patients before and after such training sessions.

In particular, in patients with diabetes we observed an increase in the use of ACR and eGFR testing, allowing to update the detected prevalence of CKD stages 3A to 5 in this cohort, which increased from 6.3 to 12.7% (+ 102%). Likewise, in patients with hypertension, this latter prevalence increased from 5.6 to 9.9% (+ 77%) and in those with heart failure it went from 10.8 to 23.7% (+ 119%). On one hand, these results, which focus on moderate to severe CKD, are highly relevant, as these patients would certainly benefit from the referral to a nephrologist. On the other hand, the encouraging data in the overall population regarding the use of ACR (+ 121%) and eGFR (+ 43%) allow the early identification of patients at risk.

In this regard, it is now consolidated that treatments for CKD initiated at early stages are more effective [25–27]. Testing for CKD is accepted by the population due to its low-cost and accuracy and, actually, individuals with CKD express a preference for early communication about a CKD diagnosis [28].

Screening and treatment in earlier stages could occur in primary care practices or community-based settings. In fact, there are clear guidelines for CKD treatment, and the natural history of the disease, including consequences of inadequate treatment, are well known [2]. As for Vanholder R et al. [29], high savings for the national health care system are achieved through prevention and by slowing down the progression of the disease by means of early detection and intervention, focusing on GP training and CKD awareness in primary care [30].

Therefore, it would be of great importance to increase our attention towardsthe early detection of CKD. The importance of raising awareness about the management of CKD among general practitioners and specialists alike cannot be underestimated, particularly as more therapeutic options are becoming available for these patients.

With regard to study limitations, in this POC study we have to highlight the fact that only a small cohort of Apulian GPs with a Millewin platform installed in their management systems were selected and that the ACR and eGFR tests were undertaken by different laboratories scattered across Apulia region, hence potentially inducing selection and sensibility/specificity biases respectively for patients and laboratory tests (increased variability of their results). For further studies, it would definitely be useful to collect more data with regard to the GPs participating in the study in order to check for potential confounders (e.g. GPs’ age, experience, location/deprivation index of the municipalities where they work and availability/distance from laboratories able to undertake standard assays), exploiting a randomized control study design.

Furthermore, given the small scale of this POC study and the data we collected, it still remains unclear whether this interventional strategy is sustainable over time and financially by health care systems. The last health technology assessment aspect will be thoroughly evaluated in a separate and large scale (national level) study by our research group.

Therefore, based on our findings, we can argue that teaching/training devoted to GPs with networking supported by specialised nephrology experts succeeds both in improving the GPs’ CKD awareness, providing vital support to prevention, and in increasing its early diagnosis and identification in at-risk categories, slowing down the progression of the disease, thus resulting in high savings as well, given the large cost gap between the early and late stages of CKD [31].

## Supplementary Information

Below is the link to the electronic supplementary material.

Supplementary file1 Supplementary Material 1: SQL Query executed in Millewin Environment to collect patients data from each GP database at T0 and T6. (TXT 8 KB)

Supplementary file2 Supplementary Material 2: Normality checks for age covariate. Pooled analysis (overall and stratified by diseases) results. (DOCX 27 KB)

Supplementary file3 Supplementary Material 3: Power analysis for effect detection details. (DOCX 12 KB)

Supplementary file4 Supplementary Material 4: Unpooled analysis (by single GP as statistical unit) results. (DOCX 24 KB)

## Data Availability

The anonymized dataset generated and analysed in this study is available from the corresponding author on request.

## Footnotes

1 https://eur-lex.europa.eu/legal-content/IT/ALL/?uri=celex:32017R0745.

## Code availability

The SQL script for data extraction from Millewin is available as a supplementary document (Supplementary material S1).
