# Supplementary material for: “The Disease Awareness Innovation Network” for chronic kidney disease identification in general practice
Source: J Nephrol. 2022 Jun 14;35(8):2057–65. doi: 10.1007/s40620-022-01353-6 (PMC9584961; doi:10.1007/s40620-022-01353-6)
Supplement: Supplementary file 2 — Supplementary file2 Supplementary Material 2: Normality checks for age covariate. Pooled analysis (overall and stratified by diseases) results. (DOCX 27 KB) [file 40620_2022_1353_MOESM2_ESM.docx]

Supplementary Material 2

# Age normality checks

list.tot.fin %>%
 sample_n_by(TIME,replace = F,size = 1500) %>%
 group_by(TIME) %>%
 shapiro_test(età)

## # A tibble: 2 x 4
## TIME variable statistic p
## <chr> <chr> <dbl> <dbl>
## 1 T0 età 0.986 8.95e-11
## 2 T6 età 0.985 1.66e-11

*# Density plot*
ggdensity(list.tot.fin$età, fill = "red")


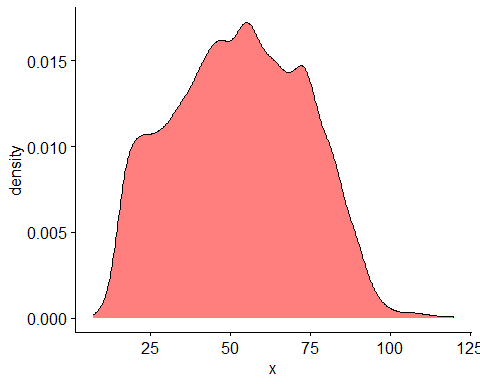


*# QQ plot*
ggqqplot(list.tot.fin$età)


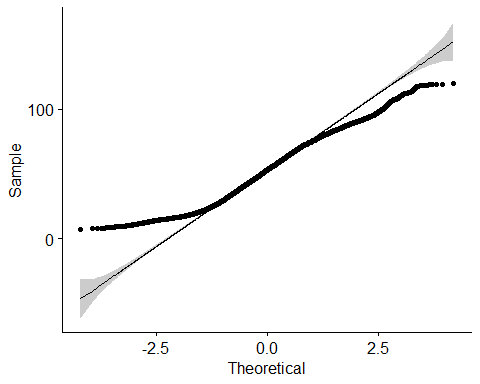


# Analysis with pooled patients T0 vs T6

## Overall KDIGO

table1(~ KDIGO_YES_NO|TIME, data=list.tot.fin,overall=FALSE,caption = "KDIGO Test Overall")

*KDIGO Test Overall*

|  | T0 (N=17854) | T6 (N=18662) |
| --- | --- | --- |
| **KDIGO_YES_NO** |  |  |
| Not_taken | 14960 (83.8%) | 14352 (76.9%) |
| Taken | 2894 (16.2%) | 4310 (23.1%) |

tabs_gps = xtabs(~KDIGO_YES_NO+TIME, data=list.tot.fin)
mcnemar_test(tabs_gps)

## # A tibble: 1 x 6
## n statistic df p p.signif method
## * <int> <dbl> <dbl> <dbl> <chr> <chr>
## 1 36516 7611. 1 0 **** McNemar test

## Overall ACR

table1(~ ACR_YES_NO|TIME, data=list.tot.fin,overall=FALSE,caption = "ACR Test Overall")

*ACR Test Overall*

|  | T0 (N=17854) | T6 (N=18662) |
| --- | --- | --- |
| **ACR_YES_NO** |  |  |
| Not_taken | 17428 (97.6%) | 17664 (94.7%) |
| Taken | 426 (2.4%) | 998 (5.3%) |

tabs_gps = xtabs(~ACR_YES_NO+TIME, data=list.tot.fin)
mcnemar_test(tabs_gps)

## # A tibble: 1 x 6
## n statistic df p p.signif method
## * <int> <dbl> <dbl> <dbl> <chr> <chr>
## 1 36516 16424. 1 0 **** McNemar test

## Overall eGFR < 60 mL/min/1.73m^2^

table1(~ eGFR|TIME, data=list.tot.fin,overall=FALSE,caption = "eGFR Test Overall")

*eGFR Test Overall*

|  | T0 (N=17854) | T6 (N=18662) |
| --- | --- | --- |
| **eGFR** |  |  |
| Over 60 | 17453 (97.8%) | 17945 (96.2%) |
| Below 60 | 401 (2.2%) | 717 (3.8%) |

tabs_gps = xtabs(~eGFR+TIME, data=list.tot.fin)
mcnemar_test(tabs_gps)

## # A tibble: 1 x 6
## n statistic df p p.signif method
## * <int> <dbl> <dbl> <dbl> <chr> <chr>
## 1 36516 16775. 1 0 **** McNemar test

# Stratified by Diseases

## Heart Failure

### Heart Failure KDIGO

list.tot.fin.hf = list.tot.fin %>% filter(list.tot.fin$`scompenso card`==1)
table1(~ KDIGO_YES_NO|TIME, data=list.tot.fin.hf,
 overall=FALSE,caption = "KDIGO Test in HF Patients")

*KDIGO Test in HF Patients*

|  | T0 (N=250) | T6 (N=245) |
| --- | --- | --- |
| **KDIGO_YES_NO** |  |  |
| Not_taken | 194 (77.6%) | 142 (58.0%) |
| Taken | 56 (22.4%) | 103 (42.0%) |

tabs_gps = xtabs(~KDIGO_YES_NO+TIME, data=list.tot.fin.hf)
mcnemar_test(tabs_gps)

## # A tibble: 1 x 6
## n statistic df p p.signif method
## * <int> <dbl> <dbl> <dbl> <chr> <chr>
## 1 495 36.5 1 0.00000000153 **** McNemar test

### Heart Failure ACR

table1(~ ACR_YES_NO|TIME, data=list.tot.fin.hf,
 overall=FALSE,caption = "ACR Test in HF Patients")

*ACR Test in HF Patients*

|  | T0 (N=250) | T6 (N=245) |
| --- | --- | --- |
| **ACR_YES_NO** |  |  |
| Not_taken | 238 (95.2%) | 194 (79.2%) |
| Taken | 12 (4.8%) | 51 (20.8%) |

tabs_gps = xtabs(~ACR_YES_NO+TIME, data=list.tot.fin.hf)
mcnemar_test(tabs_gps)

## # A tibble: 1 x 6
## n statistic df p p.signif method
## * <int> <dbl> <dbl> <dbl> <chr> <chr>
## 1 495 159. 1 1.84e-36 **** McNemar test

### Heart Failure eGFR < 60 mL/min/1.73m^2^

table1(~ eGFR|TIME, data=list.tot.fin.hf,overall=FALSE,caption = "eGFR Test in HF Patients")

*eGFR Test in HF Patients*

|  | T0 (N=250) | T6 (N=245) |
| --- | --- | --- |
| **eGFR** |  |  |
| Over 60 | 223 (89.2%) | 187 (76.3%) |
| Below 60 | 27 (10.8%) | 58 (23.7%) |

tabs_gps = xtabs(~eGFR+TIME, data=list.tot.fin.hf)
mcnemar_test(tabs_gps)

## # A tibble: 1 x 6
## n statistic df p p.signif method
## * <int> <dbl> <dbl> <dbl> <chr> <chr>
## 1 495 118. 1 1.62e-27 **** McNemar test

## Diabetes

### Diabetes KDIGO

list.tot.fin.hf = list.tot.fin %>% filter(list.tot.fin$DMT2=="Diabetes")
table1(~ KDIGO_YES_NO|TIME, data=list.tot.fin.hf,
 overall=FALSE,caption = "KDIGO Test in T2DM Patients")

*KDIGO Test in T2DM Patients*

|  | T0 (N=1932) | T6 (N=1959) |
| --- | --- | --- |
| **KDIGO_YES_NO** |  |  |
| Not_taken | 1406 (72.8%) | 1065 (54.4%) |
| Taken | 526 (27.2%) | 894 (45.6%) |

tabs_gps = xtabs(~KDIGO_YES_NO+TIME, data=list.tot.fin.hf)
mcnemar_test(tabs_gps)

## # A tibble: 1 x 6
## n statistic df p p.signif method
## * <int> <dbl> <dbl> <dbl> <chr> <chr>
## 1 3891 182. 1 1.84e-41 **** McNemar test

### Diabetes ACR

table1(~ ACR_YES_NO|TIME, data=list.tot.fin.hf,
 overall=FALSE,caption = "ACR Test in T2DM Patients")

*ACR Test in T2DM Patients*

|  | T0 (N=1932) | T6 (N=1959) |
| --- | --- | --- |
| **ACR_YES_NO** |  |  |
| Not_taken | 1724 (89.2%) | 1464 (74.7%) |
| Taken | 208 (10.8%) | 495 (25.3%) |

tabs_gps = xtabs(~ACR_YES_NO+TIME, data=list.tot.fin.hf)
mcnemar_test(tabs_gps)

## # A tibble: 1 x 6
## n statistic df p p.signif method
## * <int> <dbl> <dbl> <dbl> <chr> <chr>
## 1 3891 942. 1 7.27e-207 **** McNemar test

### Diabetes eGFR < 60 mL/min/1.73m^2^

table1(~ eGFR|TIME, data=list.tot.fin.hf,overall=FALSE,caption = "eGFR Test in T2DM Patients")

*eGFR Test in T2DM Patients*

|  | T0 (N=1932) | T6 (N=1959) |
| --- | --- | --- |
| **eGFR** |  |  |
| Over 60 | 1810 (93.7%) | 1710 (87.3%) |
| Below 60 | 122 (6.3%) | 249 (12.7%) |

tabs_gps = xtabs(~eGFR+TIME, data=list.tot.fin.hf)
mcnemar_test(tabs_gps)

## # A tibble: 1 x 6
## n statistic df p p.signif method
## * <int> <dbl> <dbl> <dbl> <chr> <chr>
## 1 3891 1375. 1 6.40e-301 **** McNemar test

## Hypertension

### Hypertension KDIGO

list.tot.fin.hf = list.tot.fin %>% filter(list.tot.fin$ipertensione==1)
table1(~ KDIGO_YES_NO|TIME, data=list.tot.fin.hf,
 overall=FALSE,caption = "KDIGO Test in HT Patients")

*KDIGO Test in HT Patients*

|  | T0 (N=5878) | T6 (N=6015) |
| --- | --- | --- |
| **KDIGO_YES_NO** |  |  |
| Not_taken | 4445 (75.6%) | 3725 (61.9%) |
| Taken | 1433 (24.4%) | 2290 (38.1%) |

tabs_gps = xtabs(~KDIGO_YES_NO+TIME, data=list.tot.fin.hf)
mcnemar_test(tabs_gps)

## # A tibble: 1 x 6
## n statistic df p p.signif method
## * <int> <dbl> <dbl> <dbl> <chr> <chr>
## 1 11893 1018. 1 2.71e-223 **** McNemar test

### Hypertension ACR

table1(~ ACR_YES_NO|TIME, data=list.tot.fin.hf,
 overall=FALSE,caption = "ACR Test in HT Patients")

*ACR Test in HT Patients*

|  | T0 (N=5878) | T6 (N=6015) |
| --- | --- | --- |
| **ACR_YES_NO** |  |  |
| Not_taken | 5535 (94.2%) | 5249 (87.3%) |
| Taken | 343 (5.8%) | 766 (12.7%) |

tabs_gps = xtabs(~ACR_YES_NO+TIME, data=list.tot.fin.hf)
*# pander(tabs_gps)*
mcnemar_test(tabs_gps)

## # A tibble: 1 x 6
## n statistic df p p.signif method
## * <int> <dbl> <dbl> <dbl> <chr> <chr>
## 1 11893 4302. 1 0 **** McNemar test

### Hypertension eGFR < 60 mL/min/1.73m^2^

table1(~ eGFR|TIME, data=list.tot.fin.hf,overall=FALSE,caption = "eGFR Test in HT Patients")

*eGFR Test in HT Patients*

|  | T0 (N=5878) | T6 (N=6015) |
| --- | --- | --- |
| **eGFR** |  |  |
| Over 60 | 5549 (94.4%) | 5417 (90.1%) |
| Below 60 | 329 (5.6%) | 598 (9.9%) |

tabs_gps = xtabs(~eGFR+TIME, data=list.tot.fin.hf)
mcnemar_test(tabs_gps)

## # A tibble: 1 x 6
## n statistic df p p.signif method
## * <int> <dbl> <dbl> <dbl> <chr> <chr>
## 1 11893 4504. 1 0 **** McNemar test
