# Supplementary material for: “The Disease Awareness Innovation Network” for chronic kidney disease identification in general practice
Source: J Nephrol. 2022 Jun 14;35(8):2057–65. doi: 10.1007/s40620-022-01353-6 (PMC9584961; doi:10.1007/s40620-022-01353-6)
Supplement: Supplementary file 3 — Supplementary file3 Supplementary Material 3: Power analysis for effect detection details. (DOCX 12 KB) [file 40620_2022_1353_MOESM3_ESM.docx]

**Supplementary Material 3**

**Tests for Two Correlated Proportions (McNemar Test)**

**Numeric Results**

────────────────────────────────────────────────────────────────────────

Solve For: Power

Power Calculation Method: Normal Approximation (Conditional)

Alternative Hypothesis: Two-Sided

────────────────────────────────────────────────────────────────────────

**Total McNemar**

**Sample Odds Prop**

**Size Diff Ratio Discord**

**Power N P10 P01 P10 - P01 P10/P01 P10 + P01 Alpha**

───────────────────────────────────────────────────────────────────────────────────────────────────────────────────────────────────

0.37876 50 0.21429 0.08571 0.12857 2.5 0.3 0.05

0.65489 100 0.21429 0.08571 0.12857 2.5 0.3 0.05

0.91907 200 0.21429 0.08571 0.12857 2.5 0.3 0.05

0.98486 300 0.21429 0.08571 0.12857 2.5 0.3 0.05

0.99964 500 0.21429 0.08571 0.12857 2.5 0.3 0.05

────────────────────────────────────────────────────────────────────────

Power The probability of rejecting a false null hypothesis when the alternative hypothesis is true.

N The total number of subjects in the study.

P10 The probability that the treatment response is 'Yes' and the standard response is 'No.'

P01 The probability that the treatment response is 'No' and the standard response is 'Yes.'

P10 - P01 The difference between P10 and P01. It is equal to the difference between Pt and Ps.

P10/P01 The McNemar odds ratio. It is not equal to the regular odds ratio between Pt and Ps.

P10 + P01 Proportion Discordant. The sum of the two off-diagonal elements, P10 and P01.

Alpha The probability of rejecting a true null hypothesis.

**Summary Statements**

────────────────────────────────────────────────────────────────────────

The comparison was made using a two-sided McNemar Test, with a Type I error rate (α) of 0.05. To detect a McNemar odds

ratio of 2.5 with a sample size of 200 pairs, the power is 0.91907. Hence, with our Millewin Data we guaranteed a sample size over such a robust threshold. The McNemar odds ratio is equivalent to a difference between two paired proportions of 0.12857 which occurs when the proportion in cell 1,0 is 0.21429 and the proportion in cell 0,1 is 0.08571. The proportion of discordant pairs is 0.3.

────────────────────────────────────────────────────────────────────────

**Tests for Two Correlated Proportions (McNemar Test)**

**Dropout-Inflated Sample Size**

────────────────────────────────────────────────────────────────────────

**Dropout-**

**Inflated Expected**

**Enrollment Number of**

**Sample Size Sample Size Dropouts**

**Dropout Rate N N' D**

────────────────────────────────────────────────────────────────────────────────────

20% 50 63 13

20% 100 125 25

20% 200 250 50

20% 300 375 75

20% 500 625 125

────────────────────────────────────────────────────────────────────────

Dropout Rate The percentage of subjects (or items) that are expected to be lost at random during the course of the study and for whom no

response data will be collected (i.e., will be treated as "missing"). Abbreviated as DR.

N The evaluable sample size at which power is computed (as entered by the user). If N subjects are evaluated out of the N'

subjects that are enrolled in the study, the design will achieve the stated power.

N' The total number of subjects that should be enrolled in the study in order to obtain N evaluable subjects, based on the

assumed dropout rate. N' is calculated by inflating N using the formula N' = N / (1 - DR), with N' always rounded up. (See

Julious, S.A. (2010) pages 52-53, or Chow, S.C., Shao, J., Wang, H., and Lokhnygina, Y. (2018) pages 32-33.)

D The expected number of dropouts. D = N' - N.

**Dropout Summary Statements**

────────────────────────────────────────────────────────────────────────

Anticipating a 20% dropout rate, 63 subjects should be enrolled to obtain a final sample size of 50 subjects.

────────────────────────────────────────────────────────────────────────

**References**

────────────────────────────────────────────────────────────────────────

Schork, M. and Williams, G. 1980. 'Number of Observations Required for the Comparison of Two Correlated

Proportions.' Communications in Statistics-Simula. Computa., B9(4), 349-357.

Machin, D., Campbell, M., Fayers, P., and Pinol, A. 1997. Sample Size Tables for Clinical Studies, 2nd Edition.

Blackwell Science. Malden, MA.

────────────────────────────────────────────────────────────────────────

**Tests for Two Correlated Proportions (McNemar Test)**

**Plots**

────────────────────────────────────────────────────────────────────────

**Procedure Input Settings**

────────────────────────────────────────────────────────────────────────

Autosave Inactive

────────────────────────────────────────────────────────────────────────

**Design Tab**

Solve For: Power

Power Calculation Method: Normal Approximation

Alternative Hypothesis: Two-Sided

Alpha: 0.05

N (Number of Pairs): 50 100 200 300 500

Probability Input Type: Off-Diagonal (P01 and P10)

P01 and P10 Input Type: McNemar Odds Ratio (P10/P01)

McNemar Odds Ratio (P10/P01): 2.5

Proportion Discordant (P10+P01): 0.3

────────────────────────────────────────────────────────────────────────
