# Supplementary material for: “The Disease Awareness Innovation Network” for chronic kidney disease identification in general practice
Source: J Nephrol. 2022 Jun 14;35(8):2057–65. doi: 10.1007/s40620-022-01353-6 (PMC9584961; doi:10.1007/s40620-022-01353-6)
Supplement: Supplementary file 4 — Supplementary file4 Supplementary Material 4: Unpooled analysis (by single GP as statistical unit) results. (DOCX 24 KB) [file 40620_2022_1353_MOESM4_ESM.docx]

Supplementary Material 4

# Analysis by General Practitioners (Unpooled Patients) T0 vs T6

## Overall KDIGO

table1(~ MEDICO|TIME*KDIGO_YES_NO, data=list.tot.fin,overall=FALSE,caption = "KDIGO Test")

*KDIGO Test*

|  | T0 | | T6 | |
| --- | --- | --- | --- | --- |
|  | Not_taken (N=14960) | Taken (N=2894) | Not_taken (N=14352) | Taken (N=4310) |
| **MEDICO** |  |  |  |  |
| GP1 | 56 (0.4%) | 4 (0.1%) | 76 (0.5%) | 15 (0.3%) |
| GP2 | 104 (0.7%) | 5 (0.2%) | 118 (0.8%) | 39 (0.9%) |
| GP3 | 169 (1.1%) | 23 (0.8%) | 253 (1.8%) | 23 (0.5%) |
| GP4 | 865 (5.8%) | 223 (7.7%) | 786 (5.5%) | 326 (7.6%) |
| GP5 | 952 (6.4%) | 166 (5.7%) | 1057 (7.4%) | 277 (6.4%) |
| GP6 | 1126 (7.5%) | 375 (13.0%) | 974 (6.8%) | 535 (12.4%) |
| GP7 | 987 (6.6%) | 122 (4.2%) | 1002 (7.0%) | 173 (4.0%) |
| GP8 | 1307 (8.7%) | 78 (2.7%) | 1282 (8.9%) | 113 (2.6%) |
| GP9 | 1154 (7.7%) | 296 (10.2%) | 1117 (7.8%) | 327 (7.6%) |
| GP10 | 753 (5.0%) | 1 (0.0%) | 783 (5.5%) | 4 (0.1%) |
| GP11 | 1143 (7.6%) | 381 (13.2%) | 881 (6.1%) | 660 (15.3%) |
| GP12 | 806 (5.4%) | 166 (5.7%) | 658 (4.6%) | 259 (6.0%) |
| GP13 | 1374 (9.2%) | 192 (6.6%) | 1262 (8.8%) | 295 (6.8%) |
| GP14 | 1099 (7.3%) | 206 (7.1%) | 886 (6.2%) | 412 (9.6%) |
| GP15 | 1059 (7.1%) | 133 (4.6%) | 1018 (7.1%) | 177 (4.1%) |
| GP16 | 1213 (8.1%) | 287 (9.9%) | 1206 (8.4%) | 289 (6.7%) |
| GP17 | 793 (5.3%) | 236 (8.2%) | 993 (6.9%) | 386 (9.0%) |

tabs_gps = xtabs(~KDIGO_YES_NO+TIME+MEDICO, data=list.tot.fin)
*# pander(tabs_gps)*
apply(tabs_gps, 3, **function**(x) mcnemar_test(x))

## $GP1
## # A tibble: 1 x 6
## n statistic df p p.signif method
## * <int> <dbl> <dbl> <dbl> <chr> <chr>
## 1 151 63.0 1 2.05e-15 **** McNemar test
##
## $GP2
## # A tibble: 1 x 6
## n statistic df p p.signif method
## * <int> <dbl> <dbl> <dbl> <chr> <chr>
## 1 266 102. 1 5.6e-24 **** McNemar test
##
## $GP3
## # A tibble: 1 x 6
## n statistic df p p.signif method
## * <int> <dbl> <dbl> <dbl> <chr> <chr>
## 1 468 190. 1 3.17e-43 **** McNemar test
##
## $GP4
## # A tibble: 1 x 6
## n statistic df p p.signif method
## * <int> <dbl> <dbl> <dbl> <chr> <chr>
## 1 2200 313. 1 4.78e-70 **** McNemar test
##
## $GP5
## # A tibble: 1 x 6
## n statistic df p p.signif method
## * <int> <dbl> <dbl> <dbl> <chr> <chr>
## 1 2452 648. 1 7.18e-143 **** McNemar test
##
## $GP6
## # A tibble: 1 x 6
## n statistic df p p.signif method
## * <int> <dbl> <dbl> <dbl> <chr> <chr>
## 1 3010 265. 1 1.33e-59 **** McNemar test
##
## $GP7
## # A tibble: 1 x 6
## n statistic df p p.signif method
## * <int> <dbl> <dbl> <dbl> <chr> <chr>
## 1 2284 687. 1 1.64e-151 **** McNemar test
##
## $GP8
## # A tibble: 1 x 6
## n statistic df p p.signif method
## * <int> <dbl> <dbl> <dbl> <chr> <chr>
## 1 2780 1064. 1 2.07e-233 **** McNemar test
##
## $GP9
## # A tibble: 1 x 6
## n statistic df p p.signif method
## * <int> <dbl> <dbl> <dbl> <chr> <chr>
## 1 2894 476. 1 1.69e-105 **** McNemar test
##
## $GP10
## # A tibble: 1 x 6
## n statistic df p p.signif method
## * <int> <dbl> <dbl> <dbl> <chr> <chr>
## 1 1541 778. 1 3.26e-171 **** McNemar test
##
## $GP11
## # A tibble: 1 x 6
## n statistic df p p.signif method
## * <int> <dbl> <dbl> <dbl> <chr> <chr>
## 1 3065 197. 1 8.08e-45 **** McNemar test
##
## $GP12
## # A tibble: 1 x 6
## n statistic df p p.signif method
## * <int> <dbl> <dbl> <dbl> <chr> <chr>
## 1 1889 293. 1 1.37e-65 **** McNemar test
##
## $GP13
## # A tibble: 1 x 6
## n statistic df p p.signif method
## * <int> <dbl> <dbl> <dbl> <chr> <chr>
## 1 3123 786. 1 6.14e-173 **** McNemar test
##
## $GP14
## # A tibble: 1 x 6
## n statistic df p p.signif method
## * <int> <dbl> <dbl> <dbl> <chr> <chr>
## 1 2603 422. 1 8.11e-94 **** McNemar test
##
## $GP15
## # A tibble: 1 x 6
## n statistic df p p.signif method
## * <int> <dbl> <dbl> <dbl> <chr> <chr>
## 1 2387 679. 1 1.14e-149 **** McNemar test
##
## $GP16
## # A tibble: 1 x 6
## n statistic df p p.signif method
## * <int> <dbl> <dbl> <dbl> <chr> <chr>
## 1 2995 564. 1 9.05e-125 **** McNemar test
##
## $GP17
## # A tibble: 1 x 6
## n statistic df p p.signif method
## * <int> <dbl> <dbl> <dbl> <chr> <chr>
## 1 2408 465. 1 3.84e-103 **** McNemar test

## Overall ACR

table1(~ MEDICO|TIME*ACR_YES_NO, data=list.tot.fin,overall=FALSE,caption = "ACR Test")

*ACR Test*

|  | T0 | | T6 | |
| --- | --- | --- | --- | --- |
|  | Not_taken (N=17428) | Taken (N=426) | Not_taken (N=17664) | Taken (N=998) |
| **MEDICO** |  |  |  |  |
| GP1 | 60 (0.3%) | 0 (0%) | 90 (0.5%) | 1 (0.1%) |
| GP2 | 109 (0.6%) | 0 (0%) | 151 (0.9%) | 6 (0.6%) |
| GP3 | 185 (1.1%) | 7 (1.6%) | 265 (1.5%) | 11 (1.1%) |
| GP4 | 1082 (6.2%) | 6 (1.4%) | 1089 (6.2%) | 23 (2.3%) |
| GP5 | 1108 (6.4%) | 10 (2.3%) | 1316 (7.5%) | 18 (1.8%) |
| GP6 | 1473 (8.5%) | 28 (6.6%) | 1452 (8.2%) | 57 (5.7%) |
| GP7 | 1096 (6.3%) | 13 (3.1%) | 1128 (6.4%) | 47 (4.7%) |
| GP8 | 1376 (7.9%) | 9 (2.1%) | 1379 (7.8%) | 16 (1.6%) |
| GP9 | 1421 (8.2%) | 29 (6.8%) | 1414 (8.0%) | 30 (3.0%) |
| GP10 | 754 (4.3%) | 0 (0%) | 787 (4.5%) | 0 (0%) |
| GP11 | 1404 (8.1%) | 120 (28.2%) | 1189 (6.7%) | 352 (35.3%) |
| GP12 | 920 (5.3%) | 52 (12.2%) | 790 (4.5%) | 127 (12.7%) |
| GP13 | 1559 (8.9%) | 7 (1.6%) | 1531 (8.7%) | 26 (2.6%) |
| GP14 | 1289 (7.4%) | 16 (3.8%) | 1245 (7.0%) | 53 (5.3%) |
| GP15 | 1138 (6.5%) | 54 (12.7%) | 1109 (6.3%) | 86 (8.6%) |
| GP16 | 1493 (8.6%) | 7 (1.6%) | 1472 (8.3%) | 23 (2.3%) |
| GP17 | 961 (5.5%) | 68 (16.0%) | 1257 (7.1%) | 122 (12.2%) |

tabs_gps = xtabs(~ACR_YES_NO+TIME+MEDICO, data=list.tot.fin)
*# pander(tabs_gps)*
apply(tabs_gps, 3, **function**(x) mcnemar_test(x))

## $GP1
## # A tibble: 1 x 6
## n statistic df p p.signif method
## * <int> <dbl> <dbl> <dbl> <chr> <chr>
## 1 151 88.0 1 6.51e-21 **** McNemar test
##
## $GP2
## # A tibble: 1 x 6
## n statistic df p p.signif method
## * <int> <dbl> <dbl> <dbl> <chr> <chr>
## 1 266 149. 1 2.86e-34 **** McNemar test
##
## $GP3
## # A tibble: 1 x 6
## n statistic df p p.signif method
## * <int> <dbl> <dbl> <dbl> <chr> <chr>
## 1 468 243. 1 9.51e-55 **** McNemar test
##
## $GP4
## # A tibble: 1 x 6
## n statistic df p p.signif method
## * <int> <dbl> <dbl> <dbl> <chr> <chr>
## 1 2200 1069. 1 1.67e-234 **** McNemar test
##
## $GP5
## # A tibble: 1 x 6
## n statistic df p p.signif method
## * <int> <dbl> <dbl> <dbl> <chr> <chr>
## 1 2452 1284. 1 2.87e-281 **** McNemar test
##
## $GP6
## # A tibble: 1 x 6
## n statistic df p p.signif method
## * <int> <dbl> <dbl> <dbl> <chr> <chr>
## 1 3010 1368. 1 1.71e-299 **** McNemar test
##
## $GP7
## # A tibble: 1 x 6
## n statistic df p p.signif method
## * <int> <dbl> <dbl> <dbl> <chr> <chr>
## 1 2284 1088. 1 1.61e-238 **** McNemar test
##
## $GP8
## # A tibble: 1 x 6
## n statistic df p p.signif method
## * <int> <dbl> <dbl> <dbl> <chr> <chr>
## 1 2780 1350. 1 1.35e-295 **** McNemar test
##
## $GP9
## # A tibble: 1 x 6
## n statistic df p p.signif method
## * <int> <dbl> <dbl> <dbl> <chr> <chr>
## 1 2894 1327. 1 1.25e-290 **** McNemar test
##
## $GP10
## # A tibble: 1 x 6
## n statistic df p p.signif method
## * <int> <dbl> <dbl> <dbl> <chr> <chr>
## 1 1541 785. 1 9.84e-173 **** McNemar test
##
## $GP11
## # A tibble: 1 x 6
## n statistic df p p.signif method
## * <int> <dbl> <dbl> <dbl> <chr> <chr>
## 1 3065 871. 1 1.64e-191 **** McNemar test
##
## $GP12
## # A tibble: 1 x 6
## n statistic df p p.signif method
## * <int> <dbl> <dbl> <dbl> <chr> <chr>
## 1 1889 645. 1 2.61e-142 **** McNemar test
##
## $GP13
## # A tibble: 1 x 6
## n statistic df p p.signif method
## * <int> <dbl> <dbl> <dbl> <chr> <chr>
## 1 3123 1508. 1 0 **** McNemar test
##
## $GP14
## # A tibble: 1 x 6
## n statistic df p p.signif method
## * <int> <dbl> <dbl> <dbl> <chr> <chr>
## 1 2603 1196. 1 4.83e-262 **** McNemar test
##
## $GP15
## # A tibble: 1 x 6
## n statistic df p p.signif method
## * <int> <dbl> <dbl> <dbl> <chr> <chr>
## 1 2387 955. 1 9.75e-210 **** McNemar test
##
## $GP16
## # A tibble: 1 x 6
## n statistic df p p.signif method
## * <int> <dbl> <dbl> <dbl> <chr> <chr>
## 1 2995 1449. 1 4.38e-317 **** McNemar test
##
## $GP17
## # A tibble: 1 x 6
## n statistic df p p.signif method
## * <int> <dbl> <dbl> <dbl> <chr> <chr>
## 1 2408 1065. 1 1.23e-233 **** McNemar test

## Overall eGFR < 60 mL/min/1.73m^2^

table1(~ MEDICO|TIME*eGFR, data=list.tot.fin,overall=FALSE,caption = "eGFR Test")

*eGFR < 60 mL/min/1.73m^2^ Test*

|  | T0 | | T6 | |
| --- | --- | --- | --- | --- |
|  | Over 60 (N=17453) | Below 60 (N=401) | Over 60 (N=17945) | Below 60 (N=717) |
| **MEDICO** |  |  |  |  |
| GP1 | 59 (0.3%) | 1 (0.2%) | 88 (0.5%) | 3 (0.4%) |
| GP2 | 109 (0.6%) | 0 (0%) | 154 (0.9%) | 3 (0.4%) |
| GP3 | 186 (1.1%) | 6 (1.5%) | 271 (1.5%) | 5 (0.7%) |
| GP4 | 1049 (6.0%) | 39 (9.7%) | 1045 (5.8%) | 67 (9.3%) |
| GP5 | 1102 (6.3%) | 16 (4.0%) | 1296 (7.2%) | 38 (5.3%) |
| GP6 | 1476 (8.5%) | 25 (6.2%) | 1453 (8.1%) | 56 (7.8%) |
| GP7 | 1085 (6.2%) | 24 (6.0%) | 1142 (6.4%) | 33 (4.6%) |
| GP8 | 1357 (7.8%) | 28 (7.0%) | 1359 (7.6%) | 36 (5.0%) |
| GP9 | 1423 (8.2%) | 27 (6.7%) | 1399 (7.8%) | 45 (6.3%) |
| GP10 | 754 (4.3%) | 0 (0%) | 786 (4.4%) | 1 (0.1%) |
| GP11 | 1485 (8.5%) | 39 (9.7%) | 1423 (7.9%) | 118 (16.5%) |
| GP12 | 955 (5.5%) | 17 (4.2%) | 874 (4.9%) | 43 (6.0%) |
| GP13 | 1542 (8.8%) | 24 (6.0%) | 1517 (8.5%) | 40 (5.6%) |
| GP14 | 1282 (7.3%) | 23 (5.7%) | 1242 (6.9%) | 56 (7.8%) |
| GP15 | 1155 (6.6%) | 37 (9.2%) | 1143 (6.4%) | 52 (7.3%) |
| GP16 | 1448 (8.3%) | 52 (13.0%) | 1446 (8.1%) | 49 (6.8%) |
| GP17 | 986 (5.6%) | 43 (10.7%) | 1307 (7.3%) | 72 (10.0%) |

tabs_gps = xtabs(~eGFR+TIME+MEDICO, data=list.tot.fin)
*# pander(tabs_gps)*
apply(tabs_gps, 3, **function**(x) mcnemar_test(x))

## $GP1
## # A tibble: 1 x 6
## n statistic df p p.signif method
## * <int> <dbl> <dbl> <dbl> <chr> <chr>
## 1 151 83.1 1 7.8e-20 **** McNemar test
##
## $GP2
## # A tibble: 1 x 6
## n statistic df p p.signif method
## * <int> <dbl> <dbl> <dbl> <chr> <chr>
## 1 266 152. 1 6.32e-35 **** McNemar test
##
## $GP3
## # A tibble: 1 x 6
## n statistic df p p.signif method
## * <int> <dbl> <dbl> <dbl> <chr> <chr>
## 1 468 252. 1 1.16e-56 **** McNemar test
##
## $GP4
## # A tibble: 1 x 6
## n statistic df p p.signif method
## * <int> <dbl> <dbl> <dbl> <chr> <chr>
## 1 2200 932. 1 1.23e-204 **** McNemar test
##
## $GP5
## # A tibble: 1 x 6
## n statistic df p p.signif method
## * <int> <dbl> <dbl> <dbl> <chr> <chr>
## 1 2452 1247. 1 4.05e-273 **** McNemar test
##
## $GP6
## # A tibble: 1 x 6
## n statistic df p p.signif method
## * <int> <dbl> <dbl> <dbl> <chr> <chr>
## 1 3010 1378. 1 1.43e-301 **** McNemar test
##
## $GP7
## # A tibble: 1 x 6
## n statistic df p p.signif method
## * <int> <dbl> <dbl> <dbl> <chr> <chr>
## 1 2284 1070. 1 1.06e-234 **** McNemar test
##
## $GP8
## # A tibble: 1 x 6
## n statistic df p p.signif method
## * <int> <dbl> <dbl> <dbl> <chr> <chr>
## 1 2780 1275. 1 2.58e-279 **** McNemar test
##
## $GP9
## # A tibble: 1 x 6
## n statistic df p p.signif method
## * <int> <dbl> <dbl> <dbl> <chr> <chr>
## 1 2894 1318. 1 1.30e-288 **** McNemar test
##
## $GP10
## # A tibble: 1 x 6
## n statistic df p p.signif method
## * <int> <dbl> <dbl> <dbl> <chr> <chr>
## 1 1541 784. 1 1.62e-172 **** McNemar test
##
## $GP11
## # A tibble: 1 x 6
## n statistic df p p.signif method
## * <int> <dbl> <dbl> <dbl> <chr> <chr>
## 1 3065 1308. 1 1.80e-286 **** McNemar test
##
## $GP12
## # A tibble: 1 x 6
## n statistic df p p.signif method
## * <int> <dbl> <dbl> <dbl> <chr> <chr>
## 1 1889 822. 1 7.37e-181 **** McNemar test
##
## $GP13
## # A tibble: 1 x 6
## n statistic df p p.signif method
## * <int> <dbl> <dbl> <dbl> <chr> <chr>
## 1 3123 1445. 1 4.36e-316 **** McNemar test
##
## $GP14
## # A tibble: 1 x 6
## n statistic df p p.signif method
## * <int> <dbl> <dbl> <dbl> <chr> <chr>
## 1 2603 1173. 1 5.11e-257 **** McNemar test
##
## $GP15
## # A tibble: 1 x 6
## n statistic df p p.signif method
## * <int> <dbl> <dbl> <dbl> <chr> <chr>
## 1 2387 1035. 1 4.98e-227 **** McNemar test
##
## $GP16
## # A tibble: 1 x 6
## n statistic df p p.signif method
## * <int> <dbl> <dbl> <dbl> <chr> <chr>
## 1 2995 1295. 1 1.15e-283 **** McNemar test
##
## $GP17
## # A tibble: 1 x 6
## n statistic df p p.signif method
## * <int> <dbl> <dbl> <dbl> <chr> <chr>
## 1 2408 1182. 1 6.06e-259 **** McNemar test

|  |  | |  | |
| --- | --- | --- | --- | --- |
|  |  |  |  |  |
|  |  |  |  |  |
|  |  |  |  |  |
|  |  |  |  |  |
|  |  |  |  |  |
|  |  |  |  |  |
|  |  |  |  |  |
|  |  |  |  |  |
|  |  |  |  |  |
|  |  |  |  |  |
|  |  |  |  |  |
|  |  |  |  |  |
|  |  |  |  |  |
|  |  |  |  |  |
|  |  |  |  |  |
|  |  |  |  |  |
|  |  |  |  |  |
